# Habitual Tea Consumption Increases the Incidence of Metabolic Syndrome in Middle-Aged and Older Individuals

**DOI:** 10.3390/nu15061448

**Published:** 2023-03-17

**Authors:** Shasha Yu, Bo Wang, Guangxiao Li, Xiaofan Guo, Hongmei Yang, Yingxian Sun

**Affiliations:** 1 Department of Cardiology, First Hospital of China Medical University, 155 Nanjing North Street, Heping District, Shenyang 110001, China; 2 Department of Clinical Epidemiology, Institute of Cardiovascular Diseases, First Hospital of China Medical University, Shenyang 110001, China

**Keywords:** metabolic syndrome, tea consumption, middle-aged, old-aged, elderly, rural China

## Abstract

In middle-aged and elderly individuals, the relationship between tea consumption and incident metabolic syndrome (MetS) is still unclear. Therefore, this study intends to figure out the relationship between tea-drinking frequency and MetS in rural middle-aged and older Chinese residents. In the Northeast China Rural Cardiovascular Health Study, 3632 middle-aged or older individuals (mean age 57 ± 8, 55.2% men) without MetS were included at baseline during 2012–2013 and were followed up on between 2015–2017. Participants showing differential tea consumption frequency were divided into the following classes: non-habitual tea drinkers, occasional tea drinkers, 1–2 times/day drinkers, and ≥3 times/day drinkers. Data showed that non-habitual tea drinking was more common among women. The frequency of tea consumption was higher in ethnic groups other than Han and among singles, as well as in concurrent smokers and drinkers and individuals with primary or lower educational status. The increasing tea consumption was in line with baseline elevations in body mass index, systolic and diastolic blood pressure, high-density lipoprotein cholesterol (HDL-C), and AST/ALT ratio. Multivariate logistic regression analysis confirmed that occasional tea drinking increased the incidence of low HDL-C [OR (95% CI): 1.268 (1.015, 1.584)], high waist circumference [OR (95% CI): 1.336 (1.102, 1.621)], and MetS [OR (95% CI): 1.284 (1.050, 1.570)]. In addition, 1–2 times/day tea drinking increased the cumulative incidence of high TG [OR (95% CI): 1.296 (1.040, 1.616)], high waist circumference [OR (95% CI): 1.296 (1.044, 1.609)] and MetS [OR (95% CI): 1.376 (1.030, 1.760)]. We demonstrated that regular tea consumption is correlated with a greater incidence of metabolic disorders and MetS. Our findings may help clarify the contradictory association reported between tea drinking and MetS development in middle-aged and older residents of rural China.

## 1. Introduction

The adverse effects of a combination of cardiovascular risk factors associated with metabolic syndrome (MetS) on the cardiovascular system are higher than those of the sum of the individual risk factors [1]. MetS encompasses insulin resistance, hypertension, dyslipidemia, and abdominal obesity [2]. Long-term studies have correlated MetS with an increased frequency of cardiovascular events and cardiovascular mortality. However, the prevalence of MetS has markedly increased in recent years. In China, the prevalence rate of MetS in 2001 was 13.7%, which increased to 24.2% in 2010–2012 [3,4]. According to the Adults Treatment Panel III criteria, the International Diabetes Federation criteria, and a harmonized definition, the prevalence rate of MetS in the overall rural population increased to 41.3%, 34.2%, and 44.1%, respectively, in 2017–2018 [5]. These prevalence rates were higher than those estimated by another study (20.5%) performed in the rural areas of China [6]. In China, the proportion of aging individuals has markedly increased, which is referred to as the silver tsunami [7]. According to the statistics from the Seventh National Population Census in 2020, the proportion of individuals aged > 60 years increased from 13.26% in 2010 to 18.70% in 2020, while that of individuals aged > 65 years increased from 8.9% in 2010 to 13.5% in 2020. As the proportion of middle-aged and elderly individuals in the population has increased, the risk factors in various age groups must be quantified to mitigate and manage MetS.

The Chinese were aware of the health-promoting and therapeutic benefits of tea 4000–5000 years ago. The health benefits of tea have been recorded in old medical texts, such as Shen Nong’s Herbal Classic. Tea has become one of the most consumed beverages in China, especially by middle-aged and elderly people. Previous studies have reported that frequent consumption of green tea exerts protective effects against cardiovascular diseases and metabolic disorders [8,9,10]. Additionally, various studies have reported that tea exerts several beneficial effects, such as antioxidant, anti-inflammatory, antibacterial, anticarcinogenic, antihypertensive, neuroprotective, cholesterol-lowering, and thermogenic effects [11,12]. The consumption of tea (*Camelia sinensis* L., green or black tea) is reported to decrease the risk of developing hypertension, dyslipidemia, diabetes, obesity, and MetS [13,14,15]. However, some studies have revealed a strong correlation between regular green tea consumption and an increased risk of developing MetS [16]. Therefore, the correlation between MetS and tea is controversial. Additionally, previous studies evaluating the effects of tea have only enrolled elderly participants. As both middle-aged and elderly subjects are keen on tea consumption, the correlation between tea consumption and MetS among middle-aged and elderly Chinese adults must be elucidated. Moreover, the effects of tea on subjects from rural areas have not been previously evaluated. Socioeconomic status (low annual income and educational status) and lifestyle habits (increased physical activity) are different among the inhabitants of rural areas, which may also affect the potential correlation between tea consumption and MetS. Previously, we demonstrated that village doctor-led interventions, including improving health knowledge, recommending healthy life habits, and monitoring the blood pressure (BP) of participants, markedly improved BP management among Chinese rural populations [17]. Understanding whether habitual tea consumption should be recommended to rural middle-aged and elderly individuals as an effective complementary non-pharmacological therapeutic approach for metabolic diseases is valuable and must be studied. Therefore, this study aimed to elucidate the correlation between tea consumption frequency and MetS in middle-aged and elderly residents of rural China to examine the effects of tea consumption on patients with metabolic disorders.

## 2. Materials and Methods

### 2.1. Study Design and Participants

A community-based prospective cohort research termed the Northeast China Rural Cardiovascular Health Study (NCRCHS) was conducted in rural Northeast China. The specific sample techniques and admittance requirements have previously been described in good detail [16]. Multi-stage, stratified, and random cluster sampling were used in the study design. From three respectively directional areas of Liaoning Province, Dawa from the eastern area, Zhangwu from the southern area, and Liaoyang from the northern area were selected in the first stage. A town from each area was chosen randomly for the following phase (a total of three towns). The final step involved selecting 8–10 rural communities at random from each town. There was a total of 26 rural villages. Each village’s eligible permanent residents (35 years of age or over) were sent an invitation to participate in the research. Pregnant women, people with malignant tumors, and people with mental illnesses were ruled out from the study [18]. The current study was certified by China Medical University’s ethics committee (Shenyang, China AF-SDP-07-1, 0-01). The baseline data for our study were based on the 2012–2013 survey results, and a total of 3988 participants who did not have MetS (older than 45 years) were enrolled. They were divided into two groups according to age: a middle-age category (between 45 and 60 years) and an elderly category (older than 60 years). The cohort was followed up from 2015 to 2017 and the median follow-up was 4.66 years. As a result of missing crucial data during the follow-up period, 356 participants were excluded. Eventually, 3632 participants were enrolled, and detailed data were gathered from them.

### 2.2. Study Variables

In light clothing and wearing no shoes, subjects’ height and weight were recorded. Utilizing non-elastic tape, the waist’s circumference was measured at the umbilicus. Body mass index (BMI) was calculated using the following equation: $\frac{\mathrm{weight}\;\left(\mathrm{kg}\right)}{{\left(\mathrm{height}\right)}^{2}\;\left(m\right)}$. An electronic standardized automated manometer (HEM-907; Omron, Tokyo, Japan) was used to measure participants’ blood pressure three times while they were seated after at least five minutes of rest. Participants who had been fasting for at least 12 h had their blood drawn in the morning. Low-density lipoprotein cholesterol (LDL-C), fasting plasma glucose (FPG), high-density lipoprotein cholesterol (HDL-C), triglyceride (TG), creatinine and uric acid were examined enzymatically.

An interview utilizing a standardized questionnaire was performed to gather comprehensive data on characteristics, lifestyle and dietary components, and medical history at the baseline. Tea consumption habit was divided into the following four groups: non-habitual tea drinkers, occasional tea drinkers, 1–2 times/day drinkers, and ≥3 times/day drinkers. To assess their dietary habits, participants were asked to recollect specifics of their food consumption during the previous year. Details on the typical weekly consumption of various foods were also requested on the questionnaire. The stated intake was roughly expressed in terms of grams per week. The following scale was used to evaluate vegetable consumption: rarely = 3, less than 1000 g = 2, more than 1000 g = 1, and more than 2000 g = 0. Meat consumption, including red meat, fish, and poultry, was evaluated using the following scale: rarely = 0, less than 250 g = 1, more than 250 g = 2, and more than 500 g = 3. A distinct diet score was assigned to each individual (meat consumption score plus vegetable consumption score, which ranges from 0–6). Lower diet scores implied adherence to the Chinese diet, while higher scores, which represented a greater meat intake and a lower vegetable intake, revealed more adherence to a Westernized diet. The same formulas for generating a diet were also employed in the ATTICA study [19]. Physical activity included occupational and leisure time physical activity. A detailed description of the methods for assessing physical activity has been presented elsewhere [20]. Participants self-reported their occupational physical activity according to the following three categories: light was physically very easy, sitting office work, e.g., secretary; moderate was work including standing and walking, e.g., store assistant; and active was work including walking and lifting, or heavy manual labour, e.g., industrial work, farm work. Self-reported leisure-time physical activity was classified into three categories: low was defined as almost completely inactive, e.g., reading, watching TV, or doing some minor physical activity but not of moderate or high level; moderate was doing some physical activity more than four hours a week, e.g., walking, cycling, etc.; and high was performing vigorous physical activity more than three hours a week, e.g., running, jogging, skiing, or regular exercise in competitive sports several times a week. Occupational and leisure-time physical activities were merged and regrouped into three categories: low was defined as subjects who reported light levels of both occupational and leisure-time physical activity; moderate was defined as subjects who reported moderate or high level of either occupational or leisure-time physical activity; and high was defined as subjects who reported a moderate or high level of both occupational and leisure-time physical activity.

MetS was defined following the unifying criteria set by the consensus of some major organizations in 2009 [21]. The coexistence of any three of the following listed five risk factors confirms metabolic syndrome diagnosis. 1. Raised blood pressure (therapy with antihypertensives in a patient with a history of hypertension is an alternative indicator). 2. High fasting glucose (an alternate indicator is medication used to treat high glucose): 100 mg/dL. 3. Reduced HDL-C levels (drug therapy for reduced HDL-C is an alternative indicator): less than 40 mg/dL (1.0 mmol/L) in men; less than 50 mg/dL (1.3 mmol/L) in women. 4. Men’s elevated waist circumference is more than 90 cm; women’s elevated waist circumference is more than 80 cm (Asians, Japanese, South and Central Americans). 5. Triglyceride elevation: 150 mg/dL (1.7 mmol/L) (drug treatment for triglyceride elevation is an alternative indication).

### 2.3. Statistical Analysis

Descriptive statistics were calculated for all the variables. Categorical variables were reported as numbers and percentages. Continuous variables were reported as mean values and standard deviations. Differences among categories were evaluated using *t*-test, ANOVA, non-parameter test, or the χ^2^-test as needed. After controlling for potential confounders, logistic regression analysis was conducted in order to estimate odds ratios (ORs) and 95% confidence intervals (CIs) for the related factors of MetS. Software SPSS version 20.0 (SPSS Inc., Chicago, IL, USA) was used for the statistical analyses, and *p* values under 0.05 were regarded as statistically significant.

## 3. Results

The baseline characteristics of the enrolled participants according to sex are listed in Table 1. At baseline, the average age of the study cohort was 57.04 years. The proportion of females in the study cohort was 44.7%. Current smokers and alcohol consumers constituted 40.7% and 26.9%, respectively, of the study cohort. The number of current smokers and alcohol consumers among males was markedly higher than that among females. The proportion of married individuals among females (99.6%) was higher than that among males (98.4%). The frequency of primary and lower education among females (62.6%) was higher than that among males (47.3%). The duration of sleep varied between males and females. Females from rural areas tended to have a short sleep duration. The number of individuals with an annual income of >20,000 CNY/year among females (30.2%) was higher than that among males (28.6%). Additionally, the number of individuals involved in low levels of physical activity among females (41.6%) was higher than that among males (28.3%). The frequency of individuals with a diet score of >3 among females (40.2%) was higher than that among males (55.9%). This indicates that the frequency of individuals with western dietary habits among males was higher than that among females. Additionally, the levels of systolic blood pressure (SBP), diastolic blood pressure (DBP), fasting plasma glucose (FPG), estimated glomerular filtration rate (eGFR), body mass index (BMI), and uric acid were upregulated in males, whereas those of high-density lipoprotein-cholesterol (HDL-C) and aspartate transaminase (AST)/alanine transaminase (ALT) ratio were downregulated at baseline. Compared with those in females, the levels of SBP, FPG, DBP, BMI, and uric acid were higher and the levels of HDL-C, AST/ALT ratio, and eGFR were lower in males during follow-up. The cumulative incidence of MetS in the study cohort was 24.0% (22.2% and 25.8% in males and females, respectively; *p* = 0.203).

Table 2 shows the baseline characteristics of the study cohort according to the frequency of tea consumption. The frequency of non-habitual tea consumption among females was higher than that among males. Compared with that in the Han ethnicity (4.8%), the frequency of tea consumption was higher in ethnic groups other than Han (10.5%). The increased numbers of individuals with a diet score of >3 and smoking and alcohol consumption habits were consistent with the increased frequency of tea consumption. Furthermore, the frequency of tea consumption was high among unmarried participants. Participants with increased tea consumption frequency were less likely to have a primary and lower education. The frequency of tea consumption was inversely and significantly correlated with the sleep duration of ≤7 h/day. Similarly, the proportion of participants with an annual income of >20,000 CNY/year was the highest among those who consume tea ≥ 3 times/day. The incidence rates of low physical activity among participants with increased frequency of tea consumption was lower than that among non-habitual tea drinkers. The increased frequency of tea consumption was consistent with the increased levels of SBP, DBP, BMI, HDL-C, and AST/ALT ratio at baseline. The levels of triglyceride (TG), uric acid, and eGFR at baseline were different from those during follow-up. The SBP, DBP, and BMI levels during follow-up and baseline were positively correlated with tea consumption frequency.

**Figure 1:**
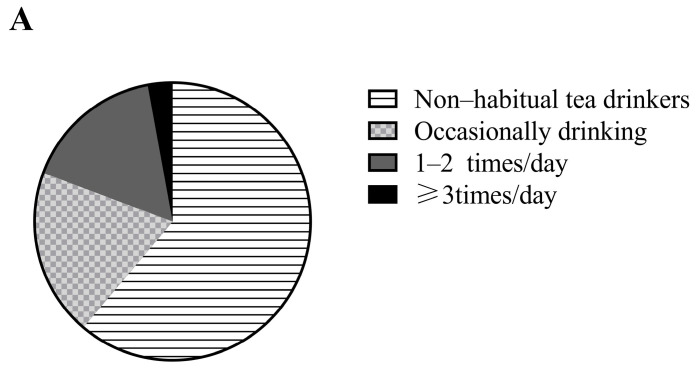

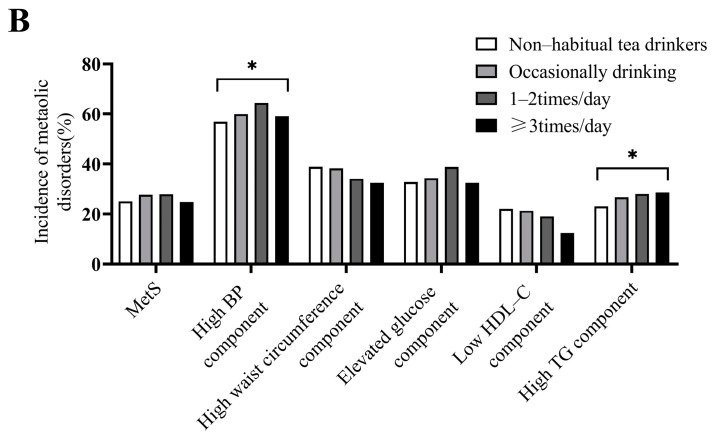
Prevalence of different frequency of tea consumption among study subjects and the cumulative incidence of metabolic disorders and metabolic syndrome in different tea consumption groups. * means *p* < 0.05 between groups. MetS: metabolic syndrome; BP: blood pressure; HDL-C: high density lipoprotein cholesterol; TG: triglyceride.

Figure 1A shows the tea consumption frequency of the participants (61.2%, 19.5%, 16.4%, and 2.9% of the participants were non-habitual tea consumers, occasional tea consumers, consumed tea 1–2 times/day, and consumed tea ≥ 3 times/day, respectively). The frequency of metabolic diseases according to tea consumption is shown in Figure 1B. The cumulative incidence of high BP and TG significantly varied depending on the frequency of tea consumption.

**Figure 2:**
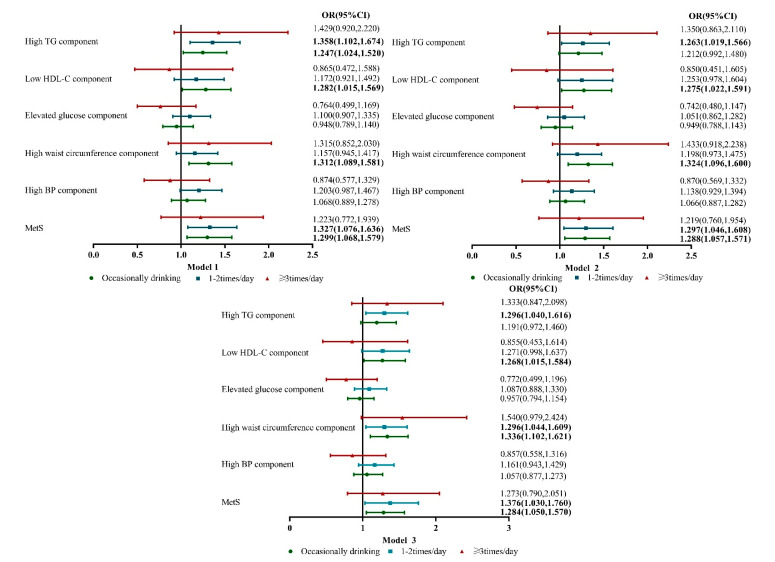
Multivariate logistic regression analysis estimated the possible relationship between tea consumption and metabolic disorders, adjusting for possible confounders. Model 1 adjusted for age and gender. Model 2 adjusted for factors included in Model 1 plus ethnicity, current drinking and smoking status, marriage status, educational status, sleep duration, annual income, and physical activity. Model 3 adjusted for factors included in Model 2 plus BMI, eGFR, uric acid, AST/ALT and diet scores.

As shown in Figure 2, multivariate logistic regression analysis after adjusting for potential confounders revealed that occasional tea consumption increased the incidence of low HDL-C [odds ratio (OR) = 1.268; 95% confidence interval (CI) = 1.015–1.584], high waist circumference [OR = 1.336; 95% CI = 1.102–1.621], and MetS [OR = 1.284; 95% CI = 1.050–1.570]. Additionally, tea consumption 1–2 times/day increased the cumulative incidence of high TG [OR = 1.296; 95% CI = 1.040–1.616], high waist circumference [OR 1.296; 95% CI = 1.044–1.609], and MetS [OR = 1.376; 95% CI = 1.030–1.760].

**Figure 3:**
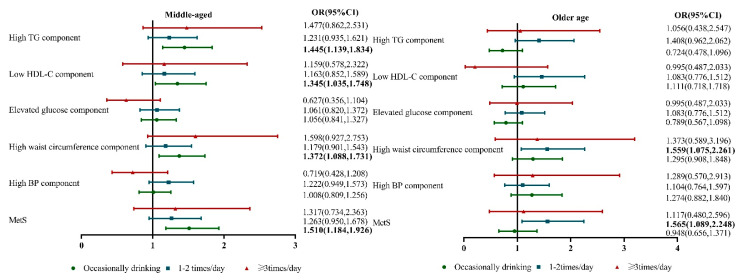
Multivariate logistic regression analysis estimated the possible relationship between tea consumption and metabolic disorders according to age categories [middle-aged group (between 45 to 60 year) and older-age group (more than 60 year)], adjusted for age, gender, race, current drinking and smoking status, marriage status, educational status, sleep duration, annual income, physical activity, BMI, eGFR, uric acid, AST/ALT and diet scores.

The study participants were subdivided into two categories according to age. As shown in Figure 3, occasional tea consumption was associated with an increased risk of developing MetS [OR = 1.510; 95% CI = 1.184–1.926], high waist circumference [OR = 1.372; 95% CI = 1.088–1.731], low HDL-C [OR = 1.345; 95% CI = 1.035–1.748], and high TG [OR = 1.445; 95% CI = 1.139–1.834] among the middle-aged group. Among the elderly group, tea consumption 1–2 times/day was correlated with an increased risk of developing MetS [OR = 1.565; 95% CI = 1.089–2.248] and high waist circumference [OR = 1.559; 95% CI = 1.075–2.261].

## 4. Discussion

This prospective study demonstrated that the risk of developing MetS among participants who occasionally consumed tea or consumed tea 1–2 times/day was higher than that among participants who non-habitually consumed tea. Additionally, the frequency of tea consumption was positively correlated with the levels of waist circumference and inversely correlated with the levels of HDL-C. These results revealed the contradicting effects of tea consumption on the metabolic health of rural middle-aged and elderly individuals.

In this study, habitual tea consumption was correlated with an increased risk of developing MetS. This is not consistent with the findings of several previous studies, which reported that tea consumption exerted beneficial effects on MetS and metabolic disorders [22,23]. Vernarelli et al. reported that *Camellia sinensis* consumption was inversely correlated with weight gain and other markers of MetS in the general population of the United States [24]. Similarly, a study conducted in a rural community in Taiwan reported that partially fermented tea consumption was inversely correlated with the incidence of MetS among elderly males, especially among those who consumed ≥240 mL of tea per day [23]. A cross-sectional study with adults from the United States revealed that the consumption of hot (brewed) tea rather than iced tea was inversely correlated with MetS [24]. In addition to findings from individual studies, systematic reviews and meta-analyses examining more than 26 randomized controlled trials (RCTs) revealed that tea consumption can alleviate the symptoms of MetS [25,26]. A systemic review of the results of eight studies demonstrated that frequent tea consumption exerts health-promoting effects and alleviates various metabolic disorders [14]. Daily consumption of green tea was demonstrated to exert beneficial health effects. Green tea is reported to inhibit lipid emulsification, suppress adipocyte differentiation, promote thermogenesis, and decrease appetite, improving systemic metabolism and decreasing fat mass [27]. Additionally, other molecules, such as theaflavins, catechins and their metabolites, and polyphenols have been implicated in the protective functions of tea against several metabolic disorders, including MetS, cardiovascular diseases, diabetes, and obesity [28]. Tea can also effectively reduce appetite, food consumption, and nutrient absorption in the gastrointestinal system, as well as alter fat metabolism [29].

In contrast to the positive impacts mentioned above, one study in England reported that the daily consumption of green tea along with high-dose caffeine did not exert therapeutic effects on metabolic disorders within 12 weeks [30]. The reasons for these conflicting results are unknown and may be related to the differential physiological and dietary conditions of different populations. According to one theory, tea extracts increase the serum uric acid levels in healthy individuals but downregulate their levels in individuals with hyperuricemia [31]. A systematic review and meta-analysis demonstrated that green tea consumption was positively correlated with the serum uric acid levels [32]. Additionally, increased uric acid levels were independently correlated with a high risk of incident MetS in the general population [33,34,35]. The potential underlying mechanism may involve uric acid-induced mitochondrial oxidative stress, which can alter AKT phosphorylation and modulate endothelial function. Moreover, uric acid is involved in the activation of the renin-angiotensin system [36,37,38,39]. According to a speculative hypothesis, the constituents of tea are complex, and some unknown constituents can upregulate the serum uric acid levels. Another possible reason may involve the dual effect of polyphenols on the serum uric acid levels. Tea extracts exert adverse effects on serum uric acid levels in healthy individuals but exert beneficial effects in individuals with hyperuricemia [31]. The findings of this study indicate that elderly and middle-aged participants who frequently consumed tea, especially those who consumed tea 1–2 times/day, were at risk to develop MetS. One study indicated that the probability of developing MetS was high among the elderly who frequently consume green tea [16]. These deleterious effects of tea consumption may be attributed to the increased excretion of urinary oxalic acid, which leads to the formation of renal stones and results in a burden on circulation [40]. However, information on tea consumption was obtained based on the recall by participants, which may have led to misclassification and information bias. This further complicates the elucidation of the correlation between tea consumption and MetS [16]. Moreover, most of these studies that confirmed the beneficial effects of tea on metabolic disorders involved participants who regularly and persistently consumed green tea or green tea extracts for ≥8–12 weeks [41,42]. Except for the regular and persistent consumption of tea or tea extracts, the amount of tea ingested should be warranted too. Recent RCTs demonstrated that the consumption of 458–886 mg of green tea catechins daily for 90 days decreased body fat in moderately overweight Chinese participants [43]. However, a recent RCT involving obese Caucasian females reported that the supplementation of 200 mg of green tea extract per day for 12 weeks did not affect various parameters, including BMI, energy and fat metabolism, insulin sensitivity, total or low-density lipoprotein (LDL)-cholesterol levels [44]. The use of small amounts of green tea extracts may not be sufficient to exert beneficial effects. Similar to the correlation between frequent tea consumption and MetS, some studies reported that the consumption of oolong and green tea increased the risk of developing type 2 diabetes (T2D) in Asian participants [45,46]. The beneficial effects of tea consumption on T2D were attributed to pesticide residues, which is supported by several epidemiological and experimental studies [46,47,48,49]. The quantity of tea consumption was correlated with the serum concentration of total organochlorine pesticides [50]. Some studies have reported a negative correlation between tea consumption and BP. For example, the consumption of green tea decoction was associated with a mild increase in BP and a marked decrease in heart rate in 76 females enrolled in an observational study [51]. Moreover, in a controversial trial with 29 elderly individuals, the consumption of green tea before lunch markedly increased SBP and DBP without affecting heart rate. Green tea was initially used to treat postprandial hypotension in elderly patients [52]. To explain tea-induced BP elevation, large amounts of green tea intake were speculated to be associated with an increased risk of developing hyperhomocysteinemia (HHcy). HHcy is demonstrated to be an independent risk factor for hypertension among residents of rural China [53,54]. The potential underlying mechanisms may involve green-tea-induced arteriolar constriction, renal dysfunction, and sodium reabsorption [55]. In addition to increasing the risk of hypertension, HHcy increases the risk of dyslipidemia. This can be partially explained by HHcy-associated hypomethylation, which results in lipid accumulation and the downregulation of the synthesis of phosphatidylcholine that is required for very low-density lipoprotein assembly and homeostasis [56]. Low HDL-C levels are attributed to the homocysteine-induced inhibition of enzymes or molecules involved in HDL particle assembly [57]. Previous studies have elucidated the correlation between HHcy, metabolic disorders, and MetS. However, the underlying mechanisms have not been elucidated. Several possible speculations have been proposed to offer some mechanistic insights. HHcy is associated with various pathological effects, including vascular damage, cytotoxicity [58], neuronal apoptosis [59], oxidative stress-induced DNA damage [60], alterations in DNA methylation [61,62], and endothelial nitric oxide production [63]. The role of homocysteine in the development of metabolic disease is unclear. Furthermore, we suggest that the contradictory findings may be attributed to the relatively high prevalence of drinking and smoking among habitual tea consumers. In China, tea consumption is a social event, and tea is the preferred drink among friends after alcohol consumption to relieve the discomforts associated with alcohol consumption. Similarly, the prevalence of smoking is high among Chinese tea consumers. These factors contributed to the increased rates of concurrent smoking and drinking among individuals consuming tea. Therefore, it must be verified if tea consumers in the study cohort constituted a small group of unhealthy subjects with a relatively high rate of smoking and alcohol consumption. These findings cannot be widely extrapolated to other populations because the study cohort was not representative. Further studies are needed to confirm the findings of this study. At baseline, the levels of SBP, DBP, BMI, and uric acid in habitual tea consumers were significantly higher than those in non-habitual tea consumers (Table 1). The combination of an unhealthy lifestyle (e.g., smoking cigarettes, drinking alcohol, and lack of leisure time exercise) and poor health status of tea drinkers relative to non-drinkers also supports this positive association [64,65,66].

In addition to poor lifestyle habits, such as smoking and drinking, dietary patterns may also be crucial factors regulating the development of MetS. Previous studies have demonstrated that the consumption of a vegetable-rich diet is associated with a decreased risk of developing metabolic disorders [67,68]. Moreover, prospective studies have reported an inverse correlation between chronic disease and plant protein intake. In contrast, the consumption of animal protein was positively correlated with the risk of developing hypertension, diabetes, and heart disease [20,69,70]. Potential mechanisms underlying these correlations have been listed below. First, the predominant amino acids vary between animal proteins and vegetable proteins. Glycine, which is predominant in animal proteins, increases BP and is associated with the risk of developing diabetes [71,72,73]. Second, the high sodium content of processed meats may be associated with hypertension and the risk of developing coronary heart disease [74,75]. In addition to high contents of sodium, nitrites in processed meats, which can be converted into nitrosamines, are toxic to pancreatic beta cells and increase the risk of diabetes in animals [76,77]. The blood nitrite concentrations can induce endothelial dysfunction [78] and insulin resistance [79] in humans. Third, advanced glycation end-products [80] or elevated inflammatory mediators [81] and gamma-glutamyltransferase [82] associated with high red meat intake can increase the risk of developing diabetes and coronary heart disease [83,84,85,86,87,88,89,90]. Finally, dietary iron, especially the heme-iron from red meat, is positively associated with diabetes, myocardial infarction, and fatal coronary heart disease [91,92,93,94,95,96,97]. Iron is responsible for catalyzing several cellular reactions, leading to the production of reactive oxygen species and induction of oxidative stress, which damages the pancreatic beta cells, increases the risk of developing diabetes, and exacerbates myocardial damage [98,99,100,101]. This study demonstrated that the increase in the frequency of tea consumption from non-habitual tea consumption to tea consumption for ≥3 times/day, western dietary habits, and increased meat consumption may affect the correlation between tea consumption and MetS. However, diet patterns were not significant factors after accounting for potential confounders. This may be related to the simplistic analysis of a diet pattern that only included the consumption of meat and vegetables. This study did not categorize animal protein into specific types because not all foods high in animal protein were equal. Red meat is positively correlated with metabolic risk factors, whereas fish and dairy are inversely correlated [102]. Further studies are needed to elucidate the relevance of dietary habits in the correlation between tea consumption and MetS.

Moreover, this study demonstrated that tea consumption was associated with MetS-related parameters (high TG, low HDL-C, and increased waist circumference). Xu et al. conducted a meta-analysis and reported that the consumption of green tea decreases the levels of LDL-C and total cholesterol but not those of HDL-C or TGs [103]. However, one systemic review of 14 unique RCTs reported that short-term (2–24 weeks) tea consumption did not significantly affect lipids in healthy or at-risk adults [104]. Recently, Huang et al. demonstrated that tea consumption delayed the aging-related decline in HDL concentrations after a six-year follow-up [19]. In this study, information on the tea consumption frequency in the past two weeks was obtained based on recall by the participants. This may have resulted in bias and consequently affected the conclusion of the study. Previous studies have indicated that the consumption of ≥3–4 cups of tea (600–900 mg tea catechins) alleviated MetS and decreased the risk of developing diabetes and cardiovascular diseases [13]. Therefore, the frequency of tea consumption may not be precise to establish the effect of tea on metabolic disorders. Several studies have demonstrated that tea consumption is frequently associated with decreased waist circumference [105]. These discrepant results may be due to the positive correlation between baseline BMI and tea consumption frequency. Tea consumption with meals decreases the absorption of non-heme iron, as tea contains tannins [106]. Black tea is reported to reduce the bioavailability of non-heme iron (Fe) by approximately 79–94%. Similarly, green tea catechins have a strong affinity for Fe because their infusions significantly reduce the bioavailability of Fe from food [11]. A recent quantitative meta-analysis examining 26 cross-sectional and case-control studies revealed that iron deficiency is significantly associated with overweight and obesity. This partially explains the reason for the positive correlation between tea consumption frequency and the risk of developing abdominal obesity [107].

Tea consumption is believed to be an effective habit with beneficial effects on hypertension, diabetes, dyslipidemia, obesity, and other metabolic disorders. Tea consumption is prevalent throughout China irrespective of rural or urban areas. However, the results of this study revealed that tea consumption increased the risk of MetS. This may be because of the increased prevalence of accompanying unhealthy lifestyle habits, such as smoking, drinking, and the increased levels of other parameters at baseline that increase the susceptibility to metabolic disorders. Therefore, non-pharmacological strategies must be carefully assessed before their application in rural inhabitants, especially middle-aged and elderly individuals.

Only middle-aged and geriatric Chinese individuals residing in Northeast China were eligible to participate in this study, which contributed to the inverse correlation between the incidence of MetS and the frequency of habitual tea consumption determined in this study. Therefore, the findings of this study may not be applicable to other populations with different demographics. Additionally, participants in this study were enrolled from rural Northeast China. This study cohort is not representative of the general population of China. Elderly individuals who frequently consumed tea in this study were a small group of non-healthy subjects with a high frequency of current smoking and alcohol consumption habits. Therefore, this is a non-representative group, which may lead to misclassification and information bias and may have distorted the effect estimates of tea consumption on MetS. Some bias was expected in the assessment because tea consumption was assessed based on self-reporting rather than through direct measurement. The potential presence of residual and unmeasured confounding factors, such as chronic illnesses, medication use, and malnutrition may increase the probability of developing MetS. Thus, the correlation reported in this study may not be accurate even though conditions were addressed for various potential confounders. This study only evaluated the frequency of tea consumption. However, information on the method used for tea preparation, the container used for tea consumption, the addition of sugar to tea, and the amount of tea consumed was not obtained. Thus, the description of the tea consumption behavior may be inaccurate. Fifth, the type of tea consumed may exert differential effects on metabolic disorders. Green tea is known to irritate the gastrointestinal tract, especially when ingested in large amounts on an empty stomach. Black tea is a gentle drink (less unpleasant) but its health benefits are limited. The composition and health benefits of dark tea, which is regarded as a mild drink, must be comprehensively investigated in the future. This study did not assess the specific types of tea consumed by the participants, which can affect the correlation between tea consumption and MetS. Additionally, comprehensive data on the intake of diets, such as dairy products, whole grains, fruits, or nuts were not available in this study. However, multivariate logistic regression analysis was performed after adjusting for some diet-related factors, including vegetable and meat consumption scores, which may partially mitigate the confounding effect of diet.

## 5. Conclusions

Our research concluded that among middle-aged and elderly rural Chinese participants, occasionally drinking and 1–2 times/day drinking tea is linked to a higher risk of developing MetS. Additional research is required to clarify the paradoxical association between tea consumption and MetS described in the literature and to identify potential mechanisms underlying the adverse effects of tea consumption.

## Figures and Tables

**Table 1:** Characteristics of study participants according to gender.

Baseline Characteristics	Total (n = 3632)	Men (n = 2005)	Women (n = 1627)	*p*-Value *
Age, years	57.04 ± 8.29	58.15 ± 8.57	55.67 ± 7.71	<0.001
Han ethnicity	3417 (94.1)	1891 (94.3)	1526 (93.8)	0.276
Current smoking (yes)	1480 (40.7)	1167 (58.2)	313 (19.2)	<0.001
Current drinking (yes)	978 (26.9)	923 (46.0)	55 (3.4)	<0.001
Married	3593 (98.9)	1972 (98.4)	1621 (99.6)	<0.001
Primary and lower education	1966 (54.1)	948 (47.3)	1018 (62.6)	<0.001
Sleep duration (h/day)				<0.001
≤7	1893 (52.2)	963 (48.1)	930 (57.3)	
7–8	991 (27.3)	575 (28.7)	416 (25.6)	
8–9	482 (13.3)	300 (15.0)	182 (11.2)	
>9	261 (7.2)	165 (8.2)	96 (5.9)	
Annual income (CNY/year)				0.025
≤5000	454 (12.5)	277 (13.8)	177 (10.9)	
5000–20,000	2111 (58.2)	1154 (57.6)	957 (58.9)	
>20,000	1064 (29.3)	572 (28.6)	492 (30.2)	
Physical activity				<0.001
Low	1234 (34.3)	562 (28.3)	672 (41.6)	
Moderate	647 (18.0)	350 (17.6)	297 (18.4)	
Severe	1719 (47.8)	1073 (54.1)	646 (40.0)	
Diet score (>3)	1776 (48.9)	1121 (55.9)	655 (40.2)	<0.001
SBP, mmHg	139.93 ± 22.76	142.57 ± 22.61	136.67 ± 22.53	<0.001
DBP, mmHg	80.65 ± 11.16	82.34 ± 11.09	78.57 ± 10.88	<0.001
BMI, kg/m^2^	23.43 ± 3.08	23.54 ± 2.85	23.31 ± 3.34	0.026
HDL-C, mmol/L	1.54 ± 0.39	1.52 ± 0.42	1.57 ± 0.35	<0.001
TG, mmol/L	1.14 ± 0.63	1.14 ± 0.67	1.14 ± 0.58	0.524
FPG, mmol/L	5.57 ± 1.06	5.67 ± 1.17	5.43 ± 0.88	<0.001
eGFR, mL/min per 1.73 m^2^	92.45 ± 13.63	92.98 ± 13.06	91.79 ± 14.27	0.009
Uric acid, μmoI/L	279.53 ± 77.18	313.33 ± 75.16	237.87 ± 56.52	<0.001
AST/ALT	1.18 (0.95, 1.43)	1.14 (0.90, 1.41)	1.22 (1.00, 1.45)	<0.001
**Follow-up Characteristics**				
SBP, mmHg	135.99 ± 21.60	139.14 ± 21.34	132.10 ± 21.29	<0.001
DBP, mmHg	80.01 ± 11.61	81.85 ± 11.68	77.74 ± 11.12	<0.001
BMI, kg/m^2^	23.64 ± 3.55	24.28 ± 3.28	22.86 ± 3.72	<0.001
HDL-C, mmol/L	1.46 ± 0.38	1.44 ± 0.39	1.48 ± 0.37	0.005
TG, mmol/L	1.41 ± 1.10	1.39 ± 1.21	1.44 ± 0.96	0.221
FPG, mmol/L	5.60 ± 1.28	5.75 ± 1.48	5.41 ± 0.95	<0.001
eGFR, mL/min per 1.73 m^2^	91.24 ± 12.24	90.55 ± 12.05	92.07 ± 12.41	<0.001
Uric acid, μmoI/L	284.10 ± 75.39	314.60 ± 73.93	246.50 ± 58.26	<0.001
AST/ALT	1.36 (1.09, 1.68)	1.33 (1.04, 1.65)	1.39 (1.14, 1.70)	<0.001
MetS	1188 (24.0)	572 (22.2)	616 (25.8)	0.203

Data are expressed as the mean ± SD or as n (%). * Student’s *t* test and χ^2^ tests were used, as appropriate, to compare differences between males and females. SD Standard deviation, CNY Chinese Yuan, SBP Systolic blood pressure, DBP Diastolic blood pressure, BMI Body mass index, HDL-C High-density lipoprotein cholesterol, TG Triglycerides, FPG Fasting plasma glucose, Metabolic syndrome.

**Table 2:** Characteristics of study participants according to tea consumption habits.

	Non-Habitual Tea Drinkers (n = 2223)	Occasional Drinkers (n = 708)	1–2 Times/Day (n = 596)	≥3 Times/Day (n = 105)	*p*-Value ^!^
**Baseline Characteristics**					
Gender (women)	1216 (54.7)	232 (32.8)	165 (27.7)	14 (13.3)	<0.001
Age, years	57.12 ± 8.32	56.43 ± 8.24	57.41 ± 8.27 ^#^	57.33 ± 7.90	0.143
Han ethnicity	2116 (95.2)	667 (94.2)	540 (90.6)	94 (89.5)	<0.001
Current smoking (yes)	749 (33.7)	322 (45.5)	335 (56.2)	74 (70.5)	<0.001
Current drinking (yes)	412 (18.5)	247 (34.9)	265 (44.5)	54 (51.4)	<0.001
Marriage status (yes)	2204 (99.1)	705 (99.6)	581 (97.5)	103 (98.1)	0.001
Primary and lower education	1290 (58.0)	345 (48.7)	289 (48.5)	42 (40.0)	<0.001
Sleep duration (h/day)					<0.001
≤7	1252 (56.4)	354 (50.2)	248 (41.6)	39 (37.1)	
7–8	545 (24.5)	199 (28.2)	207 (34.7)	40 (38.1)	
8–9	267 (12.0)	99 (14.0)	104 (17.4)	12 (11.4)	
>9	157 (7.1)	53 (7.5)	37 (6.2)	14 (13.3)	
Annual income (CNY/year)					0.001
≤5000	273 (12.3)	72 (10.2)	97 (16.3)	12 (11.4)	
5000–20,000	1279 (57.6)	414 (58.5)	363 (60.9)	55 (52.4)	
>20,000	668 (30.1)	222 (31.4)	136 (22.8)	38 (36.2)	
Physical activity					0.019
Low	803 (36.5)	226 (32.1)	177 (29.9)	28 (27.2)	
Moderate	379 (17.2)	124 (17.6)	120 (20.3)	24 (23.3)	
Severe	1021 (46.3)	353 (50.2)	294 (49.7)	51 (49.5)	
Diet score (>3)	1028 (46.2)	360 (50.8)	331 (55.5)	57 (54.3)	<0.001
SBP, mmHg	137.47 ± 21.96	139.54 ± 21.25 *	148.59 ± 24.84 ^^,#^	145.62 ± 24.30 ^&,$^	<0.001
DBP, mmHg	79.76 ± 10.84	81.15 ± 10.88 *	82.75 ± 11.87 ^^,#^	84.13 ± 12.97 ^&,$^	<0.001
BMI, kg/m^2^	23.15 ± 3.15	23.67 ± 2.94 *	24.10 ± 2.85 ^^,#^	24.04 ± 3.03 ^&,$^	<0.001
HDL-C, mmol/L	1.49 ± 0.35	1.54 ± 0.38 *	1.70 ± 0.46 ^^,#^	1.65 ± 0.52 ^&,$^	<0.001
TG, mmol/L	1.14 ± 0.62	1.20 ± 0.73	1.07 ± 0.53 ^^,#^	1.11 ± 0.56	0.006
FPG, mmol/L	5.56 ± 0.98	5.59 ± 1.02	5.54 ± 1.15	5.61 ± 1.91	0.844
eGFR, mL/min per 1.73 m^2^	90.68 ± 13.34	93.89 ± 14.58 *	97.10 ± 12.33 ^^,#^	93.81 ± 12.62 ^&,**^	<0.001
Uric acid, μmoI/L	272.72 ± 74.19	292.76 ± 84.63 *	284.84 ± 75.12 ^^^	304.36 ± 80.14 ^&,**^	<0.001
AST/ALT	1.15 (0.93, 1.40)	1.17 (0.92, 1.42)	1.25 (1.03, 1.50)	1.31 (1.00, 1.62)	<0.001
**Follow-up Characteristics**					
SBP, mmHg	135.20 ± 21.12	135.48 ± 20.73	139.26 ± 23.73 ^^,#^	137.54 ± 21.60	0.001
DBP, mmHg	79.40 ± 11.32	80.26 ± 11.05	81.65 ± 12.85 ^^,#^	81.77 ± 13.06 ^&^	<0.001
BMI, kg/m^2^	23.34 ± 3.60	24.07 ± 3.47 *	24.15 ± 3.34 ^^^	24.35 ± 3.49 ^&^	<0.001
HDL-C, mmol/L	1.46 ± 0.37	1.45 ± 0.40	1.44 ± 0.39	1.50 ± 0.42	0.437
TG, mmol/L	1.36 ± 0.94	1.48 ± 1.36 *	1.51 ± 1.33 ^^^	1.48 ± 0.94	0.007
FPG, mmol/L	5.58 ± 1.28	5.58 ± 1.19	5.69 ± 1.35 ^^^	5.62 ± 1.46	0.235
eGFR, mL/min per 1.73 m^2^	91.52 ± 12.33	91.65 ± 11.85	90.36 ± 11.97 ^^^	87.40 ± 13.47 ^&,$,^**	0.001
Uric acid, μmoI/L	274.77 ± 72.80	296.00 ± 76.47 *	299.45 ± 76.41 ^^^	314.16 ± 83.52 ^&,$^	<0.001
AST/ALT	1.36 (1.09, 1.69)	1.31 (1.05, 1.60)	1.42 (1.12, 1.71)	1.42 (1.10, 1.73)	0.001
MetS	557 (25.1)	196 (27.7)	166 (27.9)	26 (24.8)	0.359

Data are expressed as the mean ± SD or as n (%). ^!^ ANOVA test and χ^2^ tests were used, as appropriate, to compare differences between different tea consumption categories. * means *p* < 0.05 between Non-habitual tea drinkers vs. occasional drinkers. ^^^ means *p* < 0.05 between Non-habitual tea drinkers vs. 1–2 times/day. ^&^ means *p* < 0.001 between Non-habitual tea drinkers vs. ≥3 times/day. ^#^ means *p* < 0.05 between occasionally drinking vs. 1–2 times/day. ^$^ means *p* < 0.05 between occasionally drinking vs. ≥3 times/day. ** means *p* < 0.05 between 1–2 times/day vs. ≥3 times/day. SD Standard deviation. CNY Chinese Yuan, SBP Systolic blood pressure, DBP Diastolic blood pressure, BMI Body mass index, HDL-C High-density lipoprotein cholesterol, TG Triglycerides, FPG Fasting plasma glucose, MetS Metabolic syndrome.

## Data Availability

The data presented in this study are available on request from the corresponding author.
